# Metformin Attenuates the Inflammatory Response via the Regulation of Synovial M1 Macrophage in Osteoarthritis

**DOI:** 10.3390/ijms24065355

**Published:** 2023-03-10

**Authors:** Meng Zheng, Yuanli Zhu, Kang Wei, Hongxu Pu, Renpeng Peng, Jun Xiao, Changyu Liu, Xuying Sun

**Affiliations:** 1 Department of Orthopaedic Surgery, Tongji Hospital, Tongji Medical College, Huazhong University of Science and Technology, Wuhan 430030, China; zhengmeng2020@163.com (M.Z.); ysweikang@foxmail.com (K.W.); hongxu_pu@hust.edu.cn (H.P.); pengrenpeng@foxmail.com (R.P.); jun_xiao@hust.edu.cn (J.X.); liuchangyutj@outlook.com (C.L.); 2 Department of Pathology, Tongji Hospital, Tongji Medical College, Huazhong University of Science and Technology, Wuhan 430030, China; yuanli_zhutj@163.com

**Keywords:** metformin, osteoarthritis, macrophage, synovium, chondrocyte, PI3K

## Abstract

Osteoarthritis (OA), the most common chronic inflammatory joint disease, is characterized by progressive cartilage degeneration, subchondral bone sclerosis, synovitis, and osteophyte formation. Metformin, a hypoglycemic agent used in the treatment of type 2 diabetes, has been evidenced to have anti-inflammatory properties to treat OA. It hampers the M1 polarization of synovial sublining macrophages, which promotes synovitis and exacerbates OA, thus lessening cartilage loss. In this study, metformin prevented the pro-inflammatory cytokines secreted by M1 macrophages, suppressed the inflammatory response of chondrocytes cultured with conditional medium (CM) from M1 macrophages, and mitigated the migration of M1 macrophages induced by interleukin-1ß (IL-1ß)-treated chondrocytes in vitro. In the meantime, metformin reduced the invasion of M1 macrophages in synovial regions brought about by the destabilization of medial meniscus (DMM) surgery in mice, and alleviated cartilage degeneration. Mechanistically, metformin regulated PI3K/AKT and downstream pathways in M1 macrophages. Overall, we demonstrated the therapeutic potential of metformin targeting synovial M1 macrophages in OA.

## 1. Introduction

Osteoarthritis (OA), the most common inflammatory joint disease, mainly affects the diarthrodial joints [1]. It caused a growing economic burden to society. The incidence of OA is rising due to an increasingly aging population. OA is characterized by cartilage degeneration, osteophyte formation, subchondral bone remodeling, and synovial hyperplasia [2].

Increasing evidence indicates that macrophages represent a significant culprit in OA. In response to various stimuli, synovial macrophages differentiate into two phenotypes: pro-inflammatory M1 type and anti-inflammatory M2 type [3]. M1 macrophages are typically linked to the immune response to bacteria and intracellular pathogens and develop in inflammatory situations driven by Toll-like receptor (TLR) and interferon signaling [4]. TLRs regulate the expression of proinflammatory cytokines, and their interaction with HMGB1 increases insulin resistance in type 2 diabetes [5]. In both human and mouse OA synovium, M1 but not M2-polarized macrophages accumulated [6]. The degenerative process of OA is worsened by M1 macrophage-driven synovitis [7]. In comparison to osteoarthritic chondrocytes in monoculture, osteoarthritic chondrocytes cocultured with activated macrophages expressed significantly higher levels of matrix metalloproteinases (MMPs), IL-1ß, tumor necrosis factor-α (TNF-α), and interferon-γ (IFN-γ), suggesting that proinflammatory macrophages may exacerbate the abnormal matrix degradation and cytokine secretion already associated with osteoarthritic chondrocytes [8]. 

Metformin is a first-line therapy for type 2 diabetes due to its glucose-lowering actions via suppression of hepatic gluconeogenesis, enhancement of glucose uptake in muscle and adipose tissue, and the reduction in intestinal glucose absorption [9]. Multiple research groups have reported findings indicating the putative preventive benefits of metformin in OA development [10]. A favorable effect of metformin on chondroprotection, immunomodulation, and the pain reduction in knee osteoarthritis was consistently supported by pre-clinical and human trials [11]. However, how metformin attenuates cartilage degeneration is not clear. One study suggested that metformin lowered monocyte differentiation and prevented inflammation, oxidative stress, polarization, foam cell formation, and apoptosis in macrophages [12]. Others found in palmitate-stimulated bone marrow-derived macrophages (BMDMs), metformin caused a shift in the ratio of M1 macrophages to M2 macrophages [13], indicating metformin’s role in macrophage reprogramming. 

We hypothesized that metformin might reduce inflammation by regulating synovial macrophage polarization, thus further relieving synovitis and protecting chondrocytes from degeneration. To prove our hypothesis, cell culture and DMM model were used to identify whether metformin could rescue OA pathogenesis by targeting synovial macrophages. Our study uncovered the protective effects of metformin in treating OA in the way of anti-synovitis in macrophages.

## 2. Results

### 2.1. Increased M1-Polarized, but Not M2-Polarized Macrophages in Synovial Tissues from OA Patients and DMM Mice

**Figure 1:**
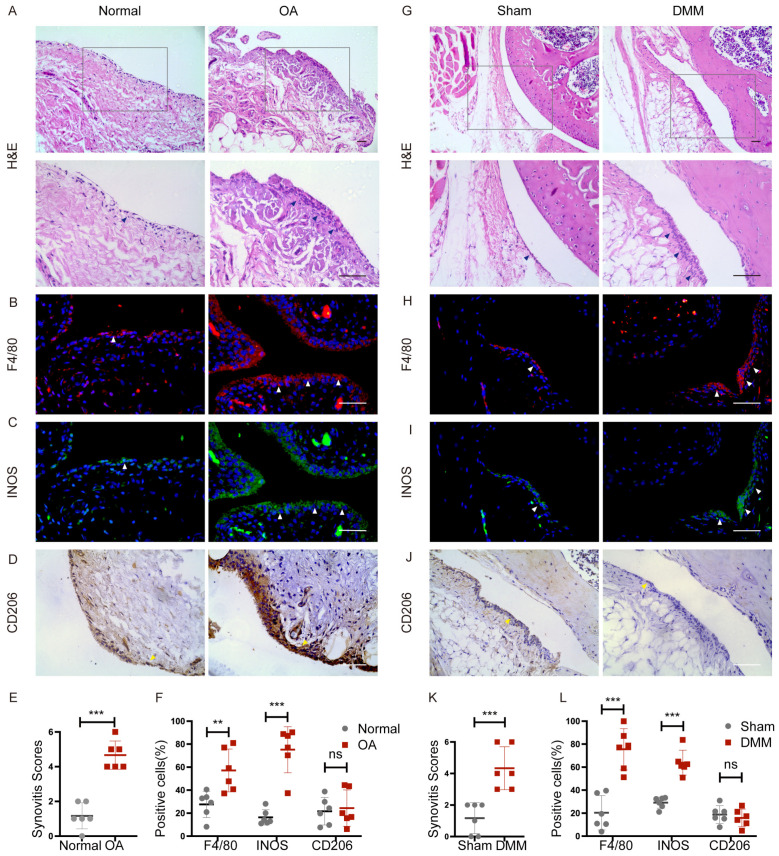
Macrophage polarization in synovium from patients with OA and DMM mice. Normal controls and OA human synovium were stained with (**A**) hematoxylin-eosin staining (H&E), immunofluorescence of (**B**) F4/80, (**C**) INOS, and immunohistochemistry of (**D**) CD206. The boxed area is enlarged in the picture below. Blue arrows showed synovial hyperplasia. White arrows indicate F4/80 and INOS-positive cells. Yellow arrows indicate CD206-positive cells. Scale bars: 50 µm. (**E**) Synovitis score quantification in normal (*n* = 6) and osteoarthritic (*n* = 6) human synovium. *** *p* < 0.001. (**F**) Quantification of the percentages of F4/80, INOS, and CD206-positive macrophages in normal and OA human synovium. ** *p* < 0.01, *** *p* < 0.001. Representative images of (**G**) H&E staining, immunofluorescence of (**H**) F4/80, and (**I**) INOS, immunohistochemistry of (**J**) CD206 in the synovium of Sham and DMM mice 2 months after surgery. Blue arrows showed synovial hyperplasia. White arrows indicate F4/80 and INOS-positive cells. Yellow arrows indicate CD206-positive cells. Scale bar: 50 µm. (**K**) Synovitis score quantification in Sham (*n* = 6) and DMM (*n* = 6) mice synovium. *** *p* < 0.001. (**L**) Quantification of the percentage of F4/80, INOS, and CD206-positive macrophages in Sham and DMM mice synovium. *** *p* < 0.01. F4/80: red; INOS: green; Nuclear: blue.

To investigate the involvement of synovial macrophages in the development of OA, we investigated the synovial inflammation and macrophage morphology changes in human normal and OA synovium. Severe synovial hyperplasia and inflammatory cell infiltration were observed in human OA synovium, with considerably higher synovitis scores than in normal samples (Figure 1A,E). The morphological characteristics of accumulated synovial macrophages were further identified via histomorphometry. Compared to controls, F4/80 (macrophage marker)-positive cells and INOS (M1-like macrophage marker)-positive cells were significantly elevated in OA synovial tissue (Figure 1B,C,F). Although the M2-like macrophage CD206 positive cell number increased, the ratio of CD206 positive macrophages did not differ in normal and OA synovium because the total cell number was also elevated in OA synovium (Figure 1D,F). These results indicated that M1-polarized macrophages, but not M2-polarized macrophages, increased in the synovium of joints in patients with OA compared to normal controls.

DMM surgery was further performed to create an OA model in mice. Similarly, no apparent cell penetration was found in the synovium of sham knees. Still, the synovium of OA knees became hyperplastic and hypertrophic, with a higher synovitis score in DMM mice (Figure 1G,K). Notably, DMM surgery reliably increased the macrophage number in synovial lining layers. The ratio of macrophages with M1-like characteristics increased, but the ratio of M2-like macrophages remained unchanged (Figure 1H–J,L). These data indicated that macrophages accumulated around OA synovium, with increased M1-like polarization, suggesting their possible significance in the pathophysiology and progression of OA.

### 2.2. Metformin Inhibits Inflammatory Cytokine Levels Produced by M1 Macrophages

**Figure 2:**
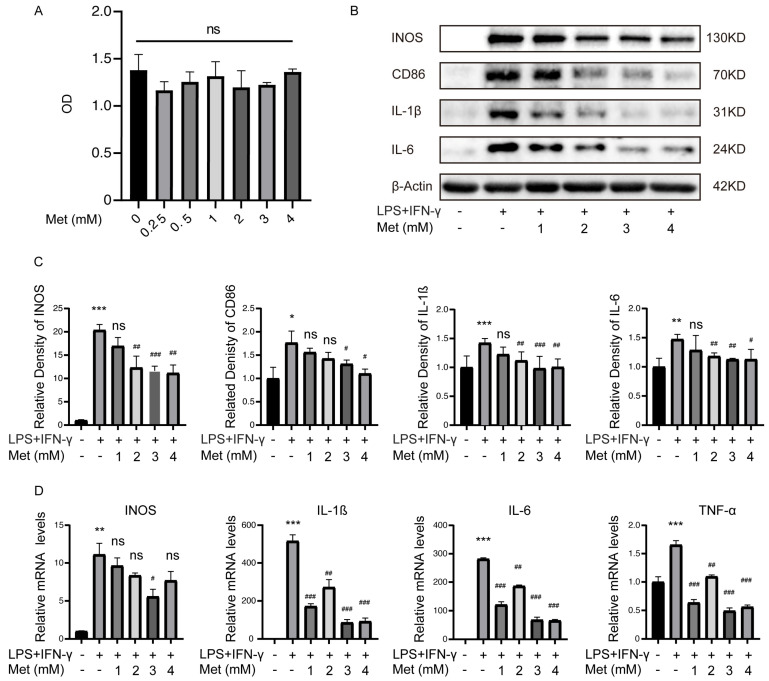
Metformin (Met) inhibits inflammatory cytokines levels produced by M1 macrophages. M1 macrophages were cultured with 0.25–4 mM metformin for 16 h. (**A**) Cell viability was determined via CCK-8 assay. ns > 0.05. (**B**) Representative Western blot and (**C**) quantitative analysis of INOS, CD86, IL-1ß, and IL-6. (**D**) mRNA levels of IL-1ß, IL-6, TNF-α, and INOS were determined via qRT-PCR. * *p* < 0.05, ** *p* < 0.01, *** *p* < 0.001 versus the vehicle-treated group. ^#^ *p* < 0.05, ^##^ *p* < 0.01, ^###^ *p* < 0.001 versus the LPS + IFN-γ treated group.

To determine if metformin alters inflammatory activities in M1 polarized macrophages, BMDMs were stimulated with lipopolysaccharide (LPS) (100 ng/mL) and IFN-γ (20 ng/mL) for 12 h and exhibited the M1 polarization phenotype [14,15,16]. Then the cells were incubated with different concentrations of metformin (Met) from 0.25 mM to 4 mM. Macrophages without intervention served as M0 control. The CCK-8 assays demonstrated that low-dose metformin has no toxic effects on BMDMs (Figure 2A). CCK-8 results also showed the viability of M1 macrophages treated with high-dose metformin (10 mM to 100 mM) was impaired from 40 mM (Figure S1A). Western blot results showed that LPS and IFN-γ stimulation without metformin treatment markedly increased the expression of the inflammatory cytokines and markers such as INOS, CD86, IL-1ß, and IL-6, consistent with the M1 phenotype of polarized macrophages. However, metformin dose-dependently reduced the expression of these cytokines and markers induced by M1-polarized macrophages, with the lowest expression at 3 mM and no longer decreased at 4 mM. (Figure 2B,C). The results from the qPCR analysis of the mRNA levels of IL-1ß, IL-6, TNF-α, and INOS were significantly elevated, and they were dose-dependently downregulated by metformin, consistent with Western blot results (Figure 2D). On the contrary, high-dose metformin enhanced the M1 polarization of macrophages and exacerbated inflammation (Figure S1B). 

### 2.3. Metformin Inhibits Macrophage-Derived CM-Induced Chondrocyte Degeneration and Cell Migration

**Figure 3:**
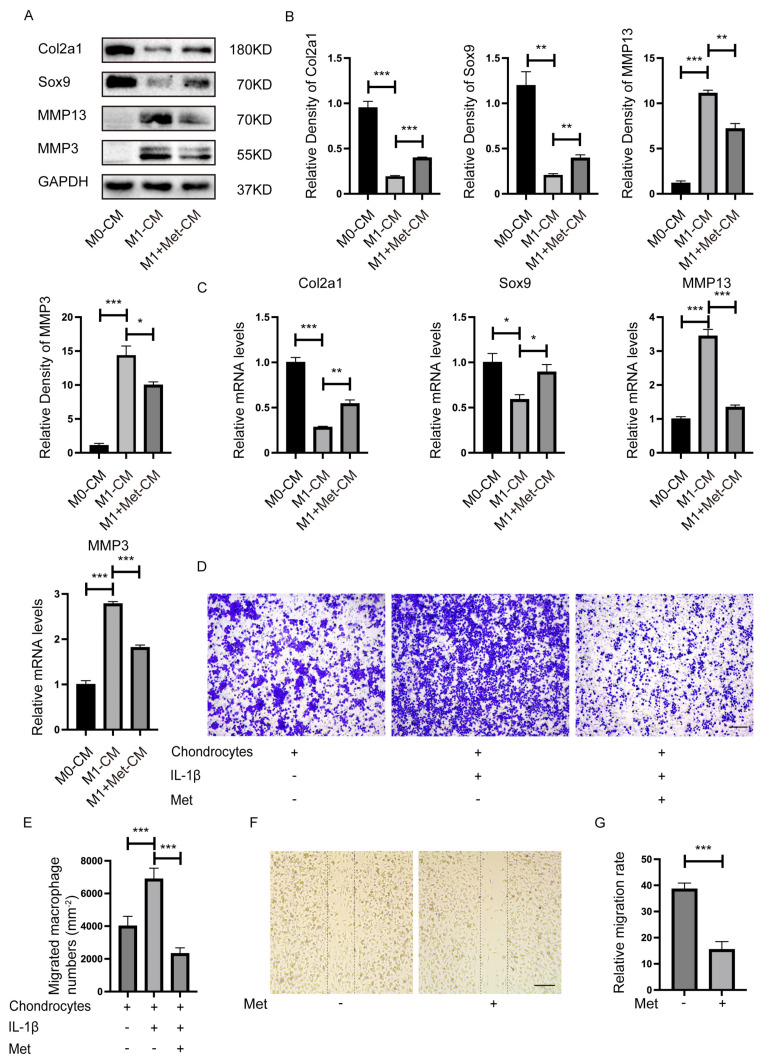
Metformin inhibits the proinflammatory effect and migration of macrophages on chondrocytes. Primary chondrocytes were cultured with CM from M0, CM (excluding LPS and IFN-γ) from M1, or CM from M1 macrophages plus metformin (3 mM) in a mixture of DMEM medium (1:1) for 24 h. (**A**) Representative Western blot and (**B**) quantitative analysis of Col2a1, Sox9, MMP13, and MMP3, and (**C**) qRT-PCR determined mRNA levels. (**D**) Representative crystal violet staining images and (**E**) quantification of migrated macrophages. (**F**) Metformin inhibited M1 macrophages migration measured using the scratch wound assay. (**G**) Quantitative analysis of the relative migration rate in (**E**) (*n* = 5). Scale bar: 100 µm. * *p* < 0.05, ** *p* < 0.01, *** *p* < 0.001.

The Western blot results demonstrated that chondrocytes cultured with M1 macrophages CM (excluding LPS and IFN-γ) increased the expression of MMP3 and MMP13, and decreased the expression of collagen type II alpha 1 (Col2a1) and SRY-Box Transcription Factor 9 (Sox9), indicating that M1 macrophages might release multiple destructive factors that contribute to cartilage degeneration in OA. However, M1-CM exposed chondrocytes in the presence of metformin for 24 h showed increased Col2a1 and Sox9 expression and decreased MMP13 and MMP3 levels compared with M1-CM group (Figure 3A,B). Results from the qPCR analysis of mRNA levels of Col2a1, Sox9, MMP13, and MMP3 were consistent with Western blot results (Figure 3C). These findings suggested that metformin reversed the inflammatory effects caused by destructive factors produced by M1 macrophages, which protected the cartilage from degeneration. 

We co-cultured BMDMs and chondrocytes using Transwell inserts to determine whether more M1 macrophages migrate to chondrocytes in inflammatory environments in vitro. BMDMs were seeded on the upper chamber and were induced to M1 macrophages by LPS + IFN-γ before the co-culture system. More M1 macrophages migrated toward chondrocytes in the presence of IL-1ß. However, metformin inhibited M1 macrophage migration toward chondrocytes caused by IL-1ß (Figure 3D,E). This phenomenon suggested that chondrocytes in the inflammatory environment might secrete certain M1 macrophage chemokines. Additionally, metformin blocked such chemotaxis. The results confirmed that metformin significantly inhibited M1 macrophage migration at 24 h in the scratch wound assay (Figure 3F,G).

### 2.4. Potential Regulatory Role of Metformin in PI3K/AKT Signaling in M1 Macrophages

**Figure 4:**
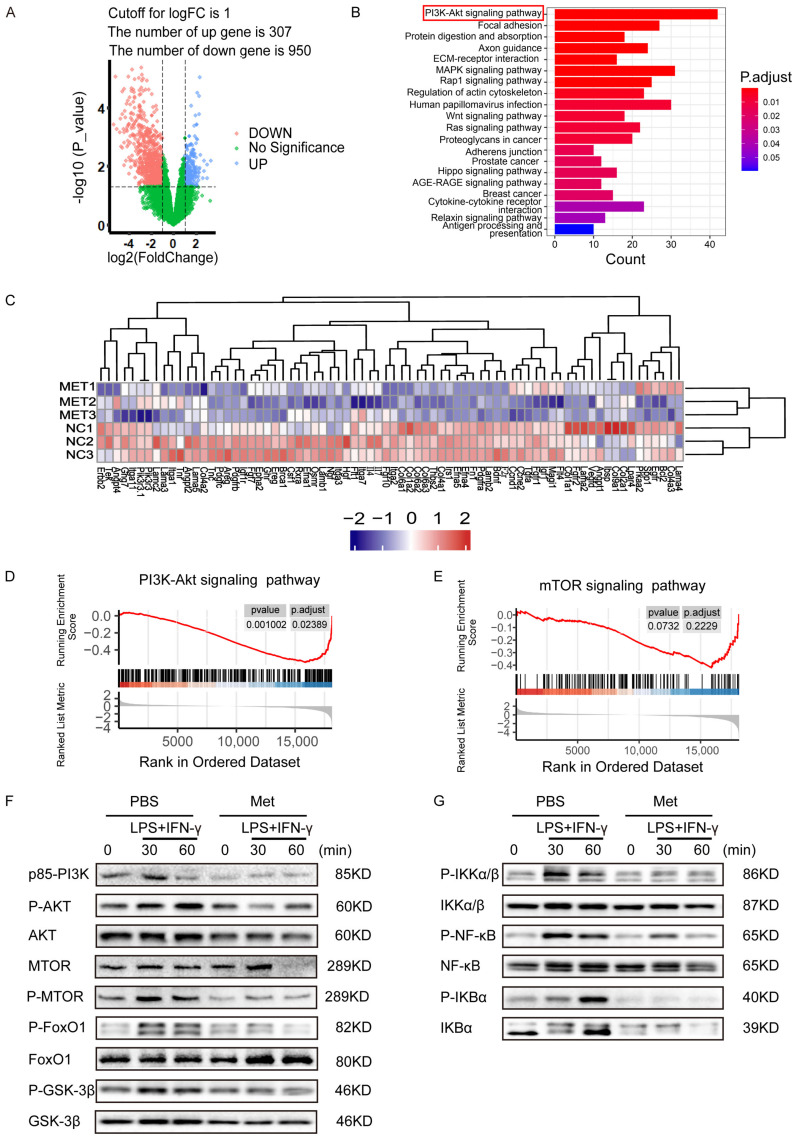
Potential regulatory role of metformin in PI3K/AKT signaling in M1 macrophages. (**A**) Volcanic Map of DEGs of metformin-treated M1 macrophages or M1 macrophages. Blue and red colors represent high and low expression values, respectively. (**B**) Representative varied KEGG pathway categories affected by metformin in M1 macrophages. The most significant enriched pathway was circled. (**C**) Heat map of DEGs of downstream genes of PI3K/AKT pathway of metformin-treated M1 macrophages or M1 macrophages. (**D**,**E**) Gene set enrichment analysis (GSEA) was used to identify the distribution of genes in the PI3K/AKT and mTOR pathway gene set of metformin-treated and control M1 macrophages groups. (**F**,**G**) Immunoblot images showing the effect of metformin on the expression of p-AKT/AKT, p-mTOR/mTOR, p-FoxO1/FoxO1, p-GSK3β/GSK3β, and signal molecules in NF-κB pathway.

To investigate the mechanism related to the anti-inflammatory and anti-migration role of metformin in M1 macrophages. Three biological replicates of M1 polarization of mouse BMDMs and three biological replicates of M1 macrophages treated with metformin (3 mM for 16 h were used to perform transcriptome analysis via high-throughput RNA sequencing (RNA-Seq). For identifying differentially expressed genes (DEGs), a *p*-value less than 0.05 was used. The final set of DEGs included genes with absolute values of logarithmic fold change (log2FC) > 1.0. Figure 4A shows that 307 DEGs were upregulated and 950 DEGs were downregulated in M1 macrophages treated with metformin compared to untreated M1 macrophages. According to kyoto encyclopedia of genes and genomes (KEGG) pathway analysis, the PI3K-AKT signaling pathway was the most significantly influenced in metformin-treated M1 macrophages (Figure 4B). PI3K/AKT pathway-related downstream genes were significantly altered as shown in heat map (Figure 4C). Additionally, the distribution of genes in several gene set pathways was displayed using gene set enrichment analysis (GSEA). Results showed that the control M1 macrophages had a high enrichment of genes relevant to the PI3K-AKT signaling pathway, pointing to a potential regulatory role for metformin in the PI3K-AKT signaling pathway (Figure 4D). The downstream mTOR pathways were also enriched, although they did not reach a significant difference (Figure 4E). The PI3K-AKT signaling and downstream mTOR, FoxO1, GSK-3ß, and IKK pathways were further tested, phosphorylation level of mTOR, FoxO1, AKT, GSK-3ß increased after LPS + IFN-γ exposure. NF-κb was also upregulated by LPS + IFN-γ, indicating a remarkable inflammation response in the presence of LPS + IFN-γ. However, mTOR, FoxO1, AKT, GSK-3ß, and NF-κb activation were notably reduced by metformin (Figure 4F,G). These results identified that metformin attenuates inflammatory response in macrophages by targeting PI3K-AKT and downstream mTOR, FoxO1, GSK-3ß, and NF-κb pathways.

### 2.5. Metformin Attenuates the Development of OA in DMM Mice

**Figure 5:**
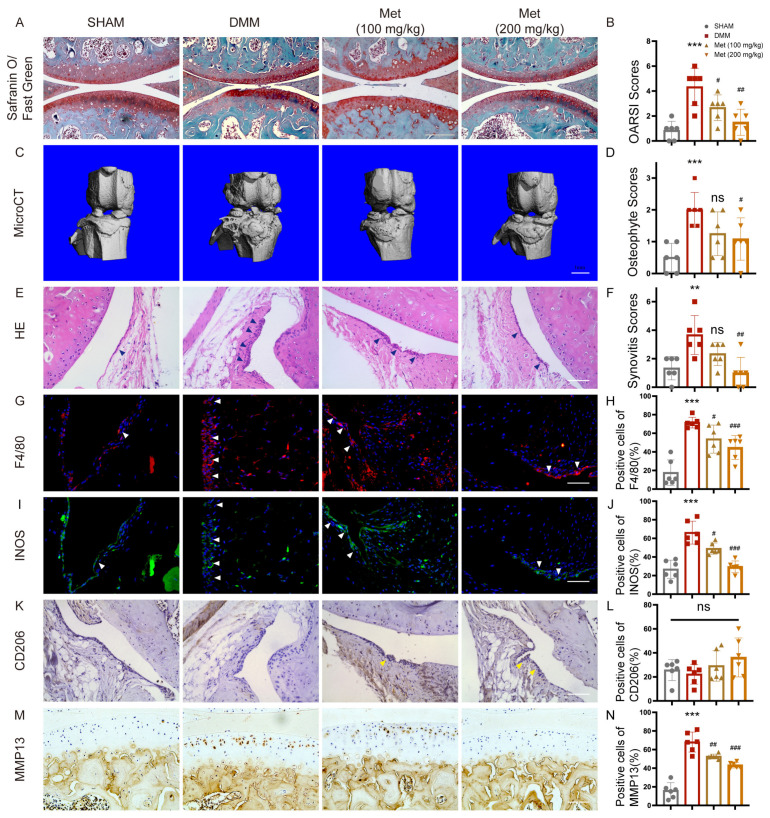
Metformin attenuates the development of OA in DMM mice. Eight-week-old mice were divided into four groups, with six in each group, including one sham-operated group. DMM surgery was performed in another three groups. Two groups of DMM mice had gavaged 100mg/kg and 200mg/kg metformin once a day, respectively, and PBS was gavaged in the remaining DMM mice. Knee joints were collected 8 weeks after surgery. (**A**) Safranin O/Fast Green staining of knee joints of each group, and (**B**) OARSI grades. (**C**) Representative microCT images of the knee joints and (**D**) osteophyte scoring. Scale bar: 1mm. (**E**) H&E staining of synovial tissues and (**F**) synovitis scoring. Representative images of immunofluorescence of (**G**) F4/80 and (**I**) INOS, immunohistochemistry of (**K**) CD206. Blue arrows showed synovial hyperplasia. White arrows indicate F4/80 and INOS-positive cells. Yellow arrows indicate CD206-positive cells. (**H**,**J**,**L**) Quantification of F4/80, INOS, and CD206-positive macrophages in mice synovial tissues. (**M**,**N**) Representative images and quantification of MMP13-positive cells in the joint cartilage. *n* = 6 per group. ** p < 0.01, *** p < 0.001 versus Sham group. *^#^ p* < 0.05, *^##^ p* < 0.01, *^###^ p* < 0.001 versus the DMM group. Scale bar: 50 µm.

We then ask whether metformin inhibits OA development in mice by targeting M1-polarized macrophages. DMM surgery was performed in mice to induce OA. DMM mice exhibited more severe cartilage degradation, as evidenced by Safranin O staining. MicroCT and HE staining showed more osteophyte formation and thicker synovial lining cells in DMM mice compared to sham-operated mice. While metformin dose-dependently reduced cartilage degradation (Figure 5A,B). Less osteophyte formation (Figure 5C,D) and milder synovitis were found after 200 mg/kg/d metformin treatment (Figure 5E,F). F4/80 and INOS-positive macrophages (inflammatory M1 type) increased, and CD206-positive macrophages (anti-inflammatory M2 type) remained unchanged after DMM surgery. Metformin dose-dependently decreased the amount of total and M1 macrophages in the synovium although the ratio of M2 type macrophages in the synovium was unchanged (Figure 5G–L). Furthermore, MMP13-positive chondrocytes were reduced in articular cartilage in both 100 mg/kg/d or 200mg/kg/d metformin-gavaged mice (Figure 5M,N). Those results confirmed that metformin attenuated cartilage degeneration by reducing synovitis through modulating macrophage reprogramming.

## 3. Discussion

Metformin usage has been linked to lower rates of mortality and cardiovascular disease in type 2 diabetes (T2D) patients with chronic kidney disease (CKD) [17]. Mineral bone disease (MBD) is a frequent consequence of chronic kidney disease (CKD) [18]. Metformin has a potential role in preventing OA in patients with CKD [19]. Long-term exposure to a diabetic environment alters bone metabolism and impairs bone microarchitecture [20]. The antidiabetic drug GLP-1 receptor agonists (GLP-1Ras) could increase BMD and decrease the implications of bone fragility [21,22]. Another antidiabetic drug, SGLT-2 inhibitors, might have detrimental effects on skeletal integrity [23]. The therapeutic effects of more antidiabetic drugs on osteoarthritis are worth further exploration.

In recent years, OA treatment has expanded to include more and more complementary therapies [24]. Routine management includes healthy exercise and weight management [25]. Non-steroidal anti-inflammatory drugs (NSAIDs), acetaminophen, duloxetine, and other medications have been licensed by the Osteoarthritis Research Society International (OARSI) to improve symptoms. Interventional treatment involves injecting corticosteroids, hyaluronic acid, and platelet-rich plasma into the affected joint. Surgical interventions, such as knee replacement and arthroscopy, are generally performed when a less invasive treatment is unsuccessful. Cell therapy uses stem cells to regenerate damaged articular cartilage. Gene therapy using genes expressing cartilage growth factor and anti-inflammatory cytokines is of interest in treating OA [26].

The understanding of the pathophysiology of OA is still changing; it has gone from being thought of as a disease that only affects cartilage, to one that affects the entire joint [1], including the synovium [27]. Macrophages play a significant role in the development of OA. High levels of macrophages are a conspicuous characteristic of inflammatory lesions, and an early sign of active OA, which exhibits an increased number of sublining macrophages in the synovium [13,28,29]. The depletion of these macrophages from inflamed tissue offers a significant therapeutic advantage. The degree of synovial macrophage infiltration correlates with the degree of joint erosion [30].

Although it was reported that metformin has therapeutic effects in treating OA, it is still unclear as to whether it affects the tissues around the joint, especially the inflammatory response in the synovium during OA development.

In the present study, we confirmed that M1-like macrophages accumulated in the synovium, both in human samples and in DMM surgical mice, consistent with previous studies [31]. M1 macrophages increased the expression of inflammatory cytokines and markers such as INOS, CD86, IL-1ß, and IL-6. At the same time, metformin could reduce these cytokine levels induced by M1 macrophages. Stimulating chondrocytes with M1 macrophages, CM increased the expression of the breakdown enzymes such as MMP13 and MMP3, while decreasing Col2a1 and Sox9 levels. The degenerative phenotypes of chondrocytes cultured with M1 macrophage CM were identical to those of an IL-1ß-treated condition. However, metformin significantly reversed these degenerative cartilage markers caused by M1 macrophages CM. Macrophages were also responsive to IL-1ß. Chondrocytes with IL-1ß treatment induced more M1 macrophages to migrate to the bottom chamber. To our surprise, metformin could inhibit such a migration of the M1 macrophages, which suggested that metformin might delay OA phenotypes by hampering M1 macrophage migration. It deserves more research.

The protective effect of metformin was further investigated in DMM mice. We found that metformin reduced the infiltration of M1 macrophages into the synovium in DMM mouse models, and alleviated synovitis and osteophyte formation, suggesting that metformin also has an indirect protective effect on chondrocytes by inhibiting the inflammatory secretion and migration of M1 macrophages. RNA-seq analysis was conducted in M1 macrophages treated with or without metformin for 16h. KEGG pathway analysis showed that the PI3K/AKT signaling pathway was markedly inhibited in metformin-treated M1 macrophages compared to the untreated group. PI3K/AKT is one of the most critical pathways that controls many cellular processes, including cell division, autophagy, survival, and differentiation [32]. AKT is activated by the phosphorylation of the transcription factor IκB kinase (IKK), a positive regulator of NF-kB, regulating the expression of genes with antiapoptotic activity [33]. Glycogen synthase kinase-3 (GSK3), the mammalian target of rapamycin (mTOR), are both AKT targets involved in protein synthesis, glycogen metabolism, and cell cycle regulation [34]. The phosphorylation of FoxO1 regulates NF-κB nuclear translocation by activating PI3K/AKT during aging [35]. Western blotting showed that changes in the downstream IKK, mTOR, NF-κB, and GSK-3ß pathways were also regulated. These results indicated that metformin could regulate many aspects of M1 macrophages through the PI3K/AKT signaling pathways.

Indeed, a lot of studies have explored the effects of macrophages on OA by regulating synovitis. However, the specific mechanism needs to be further explored. The study reconfirmed that macrophages play an important role in the development of OA synovitis. The increased infiltration of M1 macrophages is accompanied by the aggravation of OA symptoms. Metformin can significantly inhibit the infiltration of M1 macrophages in OA. This finding has been rarely reported so far, which further provides the basis for metformin in the treatment of OA.

There are many limitations in our study. First, although metformin attenuated M1 macrophage migration, we cannot exclude the direct role of metformin on chondrocytes in vivo. The protective effect of metformin may probably consist of the direct role in chondrocytes, and the indirect part in macrophage migration in the synovium, which significantly inhibited the inflammatory response in the tissue around the joint. Second, the precise mechanism underlying how metformin affects macrophage migration still needs further exploration. AMP-activated protein kinase (AMPK) has a significant role in its mechanism of action. Is the AMPK pathway also related to PI3K/AKT signaling? Despite the PI3K/AKT pathways, STAT6 was also vital in regulating macrophage polarization [36]. We will examine the exact mechanisms in our future studies.

## 4. Materials and Methods

### 4.1. Reagents and Antibodies

Recombinant murine IL-1β and recombinant murine M-CSF protein were purchased from R&D Systems (Minneapolis, MN, USA). Recombinant murine IFN-γ was purchased from Peprotech (Rocky Hill, NJ, USA). Lipopolysaccharides (LPS) were obtained from Biosharp (Beijing, China). Metformin hydrochloride was purchased from CTAO (Eugene, OSU, USA). Anti-p85-PI3K, MTOR, p-MTOR, FoxO1, p-FoxO1, AKT, p-AKT, GSK3ß, p-GSK3ß, NF-κB, p-NF-κB, IκBα, p-IκBα, IκB kinase (IKK)-β, and p-IKKα/β antibodies were supplied by Cell Signaling Technology (Beverly, MA, USA). MMP3, MMP13, INOS, and CD206 antibodies were obtained from Abcam (Cambridge, UK). Col2a1 and Sox9 antibodies were purchased from Abclonal (Wuhan, China). IL-1ß, IL-6, CD86, and F4/80 antibodies were purchased from Proteintech (Wuhan, China). GAPDH, ß-Actin, and secondary antibodies were purchased from Boster (Wuhan, China).

### 4.2. Human Synovial Sample Collection

Normal human synovium tissues were collected from six objects injured in car accidents, and who had no history of arthritic diseases (five men and one woman, aged 45 ± 2.8 years old). Human OA synovium tissues were collected from six objects who underwent knee replacement surgery (two men and four women, average age 64.67 ± 5.87 years old). People with cancer, diabetes or other severe diseases in the last 5 years were excluded. The collection of human samples was approved by the Ethics Committee of the Tongji Hospital (TJ-IRB20210127). Informed written agreement was obtained before the collection.

### 4.3. Isolation and Culture of Primary Chondrocytes

After the carbon dioxide euthanasia of 5-day-old neonatal mice, knee articular cartilage was dissected. Then, the articular cartilage was cut into small pieces using scissors, followed by 0.25% trypsin digestion for 30 min, and then further digested with 0.25% collagenase II dissolved in DMEM/F12 (Hyclone, Logan, UT, USA) growth media for 6–8 h at 37 °C. After centrifuging the digested chondrocytes (1500 rpm for 5 min), the supernatant was discarded, and the resuspended cells were cultured in DMEM/F12 supplemented with 10% fetal bovine serum (FBS) (Gibco, Carlsbad, CA, USA) at 37 °C in an incubator.

### 4.4. Culture of Bone Marrow-Derived Macrophages (BMDMs) and the Induction of Macrophage Polarization

Bone marrow cells were flushed out from the long bones of the mice with α-modified Eagle’s medium (α-MEM; Hyclone, Logan, UT, USA). Then, cells were cultured in α-MEM/10% FBS supplemented with 30 ng/mL M-CSF for 24 h. The non-adherent macrophages were cultured for another 3 days in the same medium. Concentrations of 100 ng/mL LPS and 20 ng/mL IFN-γ were used to induce M1 macrophage polarization. Western blot and qPCR were used to confirm the polarization phenotypes of the macrophages.

### 4.5. Protein Extraction and Western Blot

Macrophages administered with metformin (0.25–4 mM), and chondrocytes cultured with CM from macrophages were lysed for 30 min in ice-cold RIPA lysis buffer (Boster, Wuhan, China) with 1% protease and phosphatase inhibitor. The cell lysates were then immediately configured for 30 min at 10,000× *g* at 4 °C. Proteins of equal concentrations were put onto SDS-PAGE (10–15%) for electrophoresis before being transferred to polyvinylidene fluoride (PVDF) membranes. Each membrane was first blocked with 5% non-fat milk in TBST (Tris-buffered saline with 0.1% Tween 20 buffer) for 1 h, and then primary antibodies were incubated overnight at 4 °C on a shaker. Proteins were detected using an enhanced chemiluminescence kit (Thermo Fisher Scientific, Waltham, MA, USA) in the ChemiDoc XRS System after being incubated with horseradish peroxidase-conjugated secondary antibodies for 1 h (Bio-Rad Laboratories, Hercules, CA, USA).

### 4.6. RNA Isolation and qPCR

Total RNA was extracted from cells using Trizol (Takara, Otsu, Shiga, Japan) according to the manufacturer’s instructions, and cDNA was synthesized using the HiScript II Q RT Supermix for quantitative polymerase chain reaction (qPCR) (Vazyme, Nanjing, China). Next, qPCR was performed with ChamQ SYBR Color qPCR (Vazyme, Nanjing, China). All the data were normalized to GAPDH. The primer sequences used are listed below: GAPDH, 5′-ACGGGAAGCTCACTGGCATGGCCTT-3′ (sense), 5′-CATGAGGTCCACCACCCTGTTGCTG-3′ (antisense); IL-1ß, 5′-GAAATGCCACCTTTTGACAGTG-3′ (sense), 5′-TGGATGCTCTCATCAGGACAG-3′ (antisense); IL-6, 5′-TTCACAAGTCGGAGGCTT-3′ (sense), 5′-CAGTTTGGTAGCATCCAT-3′ (antisense); TNF-α, 5′- CAGGCGGTGCCTATGTCTC-3′ (sense), 5′- CGATCACCCCGAAGTTCAGTAG-3′ (antisense); INOS, 5′-CAGGAGGAGAGAGATCCGATTTA-3′ (sense), 5′-GCATTAGCATGGAAGCAAAGA-3′ (antisense); CD86, 5′-TCAATGGGACTGCATATCTGCC-3′ (sense), 5′-GCCAAAATACTACCAGCTCACT-3′ (antisense); Col2a1, 5′-CTTCACAGCGGTAGATCCCAG-3′ (sense), 5′-ACCAGGGGAACCACTCTCAC (antisense); MMP3, 5′-ACTCCCTGGGACTCTACCAC-3′ (sense), 5′-GGTACCACGAGGA CATCAGG-3′ (antisense); MMP13, 5′-TGATGGACCTTCT GGTCTTCTGG-3′ (sense), 5′-CATCCACATGGTTGGGAAGTTCT-3′ (antisense); Sox9, 5′-CAGCCCCTTCAACCTTCCTC-3′ (sense), 5′-TGATGGTCAGCGTAGTCGTATT-3′ (antisense). The 2^−ΔΔCq^ method was used to determine the relative mRNA levels of the target genes.

### 4.7. Cell Viability Assay

BMDMs were seeded onto 96-well plates for M1 polarization, and subsequently stimulated for 24 h with metformin. Cell proliferation was assessed using the Cell Counting Kit-8 Assay (CCK-8, TargetMol, Wellesley Hills, MA, USA). Cells were incubated for 1 h in CCK-8 media to determine the proliferation rate, and their absorbance at 450 nm was measured using a microplate reader (BioTek, Winooski, VT, USA).

### 4.8. Collection of Conditional Medium (CM)

BMDMs were seeded on 6-well plates at a density of 2 × 10^5^ cells per well. M1 polarization was induced as described above. Cultures were treated with 3 mM metformin. After 12 h of M1 induction, cells were washed entirely with phosphate-buffered saline (PBS) three times, and cultured in 2 mL of serum-free DMEM for an additional 12 h. This step removed LPS and IFN-γ from CM. The CM was collected and centrifuged for 5 min at a speed of 1000× *g*. After that, the supernatant was aliquoted and kept at minus 80 °C until it was used as CM mixed with DMEM/F12 (1:1) to treat chondrocytes.

### 4.9. Transwell Assay

The cell migration test was carried out utilizing 24-well transwell inserts with Polycarbonate (PC) membrane with 8.0 μm holes (Corning, NY, USA). Briefly, 2 × 10^4^ BMDMs suspended in 0.1 mL of serum-free media were seeded onto the top chambers with M1 polarization induction. A total of 1 × 10^5^ primary chondrocytes were seeded on the lower chambers. After a 24 h incubation, cells that remained on the top chamber were removed using cotton swabs, while cells that had migrated to the other side of the transwell were fixed with 4% paraformaldehyde (PFA) for 10 min and stained with 1% crystal violet solution. Images were captured using an inverted microscope (Eclipse TS100, Nikon, Japan). The migration was evaluated by counting the number of cells that traveled through the membranes in 5 randomly chosen areas of each transwell.

### 4.10. RNA Sequencing and Gene Set Enrichment Analysis

Total RNA was extracted from M1 macrophages treated with or without metformin (3mM). RNA purity was evaluated using the NanoDrop™ One/OneC (Thermo Scientific, Waltham, MA, USA). RNA quantification was detected using the Qubit™ RNA HS Assay Kit (Thermo Scientific, Waltham, MA, USA). RNA integrity was assessed using the Agilent 4200 TapeStation (Agilent Technologies, Palo Alto, CA, USA). Then, the libraries were constructed using the TruSeq Stranded mRNA LT Sample Prep Kit (Illumina, San Diego, CA, USA) according to the manufacturer’s instructions. AMPure XP beads (Beckman Coulter, Bria, CA, USA) were used to purify the fragments during library generation. These libraries were sequenced on an Illumina PE150, and 125 bp/150 bp paired-end reads were generated. Transcriptome sequencing and analysis were conducted by the Wuhan Biobank institution (Wuhan, China). Then, the clean reads were mapped to the reference genome using Hisat2. The FPKM of each gene was calculated using Cufflinks. HTSeqcount obtained the read counts of each gene. Expression analysis was performed using the DESeq R package. Hierarchical cluster analysis was performed to demonstrate the expression pattern of genes in different groups. KEGG pathway enrichment and GSEA analysis were performed using R, based on the hypergeometric distribution.

### 4.11. Dissociation of the Medial Meniscus (DMM) Surgery and Animal Gavage

Eight-week-old C57/BL6 male mice (*n* = 24) purchased from Gempharmatech Company (Jiangsu, China) were used for our in vivo experiment. Mice were housed in the animal facility of Tongji Hospital, with less than five mice per cage, and free access to food and water. The surgical process was approved by The Ethics Committee of Tongji Hospital. DMM was performed to induce OA, as previously reported [37]. Before the surgery, the mice were anesthetized, and the right knee was cleaned and shaved. Then, the joint capsule was opened, and the fat pad was removed so that the medial meniscus-tibial ligament could be seen under a microscope. The ligament was cut before stitching up the joint capsule and skin wound. Mice were randomly divided into four groups: (1) Sham group: a sham operation involves cutting on the right side of the knee without damaging the joint capsule [38]. (2) DMM surgery group. (3) Metformin gavaged at a dosage of 100 mg/kg/d post-surgery, or (4) Metformin gavaged at a dosage of 200 mg/kg/d for 8 weeks post-surgery.

### 4.12. Micro-Computed Tomography (MicroCT) Imaging

MicroCT analysis was performed on knees from sham-operated and DMM surgical mice using a VivaCT 40 scanner at 15 μm resolution, 70 kVP, and 112 μA X-ray energy (Scanco, Wangen-Brüttisellen, Switzerland). Following the manufacturer’s recommendations, the three-dimensional images were rebuilt. The periarticular osteophytes were chosen as the region of interest (ROI). On the medial and lateral sides of the tibia and femur, the ROI size was computed blindly on all four knee condyles (0–3), and the average was used for statistical analysis.

### 4.13. Safranin O–Fast Green, and H&E

Mouse knee joint specimens were collected immediately after scanning. After decalcification, dehydration, and paraffin embedding of the samples, tissues were cut into 5-μm sections and stained using a Safranin O and Fast Green staining kit (Solarbio, Beijing, China), and hematoxylin and eosin solutions (H&E; Servicebio, Wuhan, China), as previously described [39]. ImageJ (version 1.53k) was used to determine and to measure the Safranin O-positive cartilage areas. H&E staining was used to evaluate synovial activation by scoring synovial lining cell thickness (0–3); the medial and lateral compartments of the joint were scored separately, and the sum of the two scores was presented (maximum site score 6) [40]. Cartilage degeneration was graded in Safranin-O/Fast Green-stained sections using a 0–6 semi-quantitative scoring system [41]. Two blinded, independent graders assessed each section, and the average score was used for statistical analysis.

### 4.14. Immunohistochemistry and Immunofluorescence

After deparaffinization and hydration, sections were immersed in citrate buffer (10 mM citric acid, pH 6.0) overnight at 60 °C to unmask the antigen. A 3% hydrogen peroxide was used to inhibit endogenous peroxidase activity before immunohistochemical staining. The sections were incubated with primary antibodies overnight at 4 °C after being blocked with 3% goat serum at 37 °C for 1 h. For immunohistochemistry, the sections of horseradish peroxidase-conjugated secondary antibodies were stained for immunohistochemical staining. Next, the chromogen was observed using 3, 3-diaminobenzidine, and the sections were counterstained with hematoxylin. For immunofluorescence, sections were stained with Alexa 488- or Alexa 594-dyed secondary antibodies after primary antibodies incubation. DAPI (Boster, Wuhan, China) was utilized to label the nuclei. A fluorescence microscope (EVOS FL Auto, Thermo Fisher Scientific, Waltham, MA, USA) was used to acquire the images. Three areas of synovium were chosen for high-magnification imaging, and a mean value was determined for the positive staining of synovial intimal lining macrophages.

### 4.15. Scratch Assay

A 10 mL pipette was used to scratch the M1 macrophage monolayer in a 6-well plate. Before taking the photos, the cells were given one wash to eliminate the suspension cells. Then, the culture medium was supplemented with or without 3 mM metformin. The scratches were imaged once more at the same location after 24 h to assess cell migration. Picture J was used to gauge the width of the marks. The scratch closure rates were determined using the formula (width 0 h − width 24 h)/width 0 h × 100%.

### 4.16. Statistical Analysis

All experimental data were independently repeated at least three times. All data were presented as mean ± SEM. Student’s *t*-test was used to assess differences between two groups, while one-way analysis of variance (ANOVA) and Tukey’s multiple comparison tests were used to investigate differences between three groups using GraphPad Prism v. 8.0 (Graphpad Software Inc., San Diego, CA, USA) software. All statistical tests were two-sided. The significance level was set at *p* < 0.05.

## 5. Conclusions

Metformin attenuates the migration of M1 macrophages to the chondrocytes in vitro, and diminishes the infiltration of M1 macrophages in OA synovial tissues. Metformin plays a great therapeutic role in OA targeting M1 macrophages.

## Data Availability

The dataset of the present study is available from the corresponding author upon request.

## Supplementary Materials

The following supporting information can be downloaded at: https://www.mdpi.com/article/10.3390/ijms24065355/s1.
