# Supplementary figures and images for: Metformin Attenuates the Inflammatory Response via the Regulation of Synovial M1 Macrophage in Osteoarthritis
Source: Int J Mol Sci. 2023 Mar 10;24(6):5355. doi: 10.3390/ijms24065355 (PMC10049635; doi:10.3390/ijms24065355)

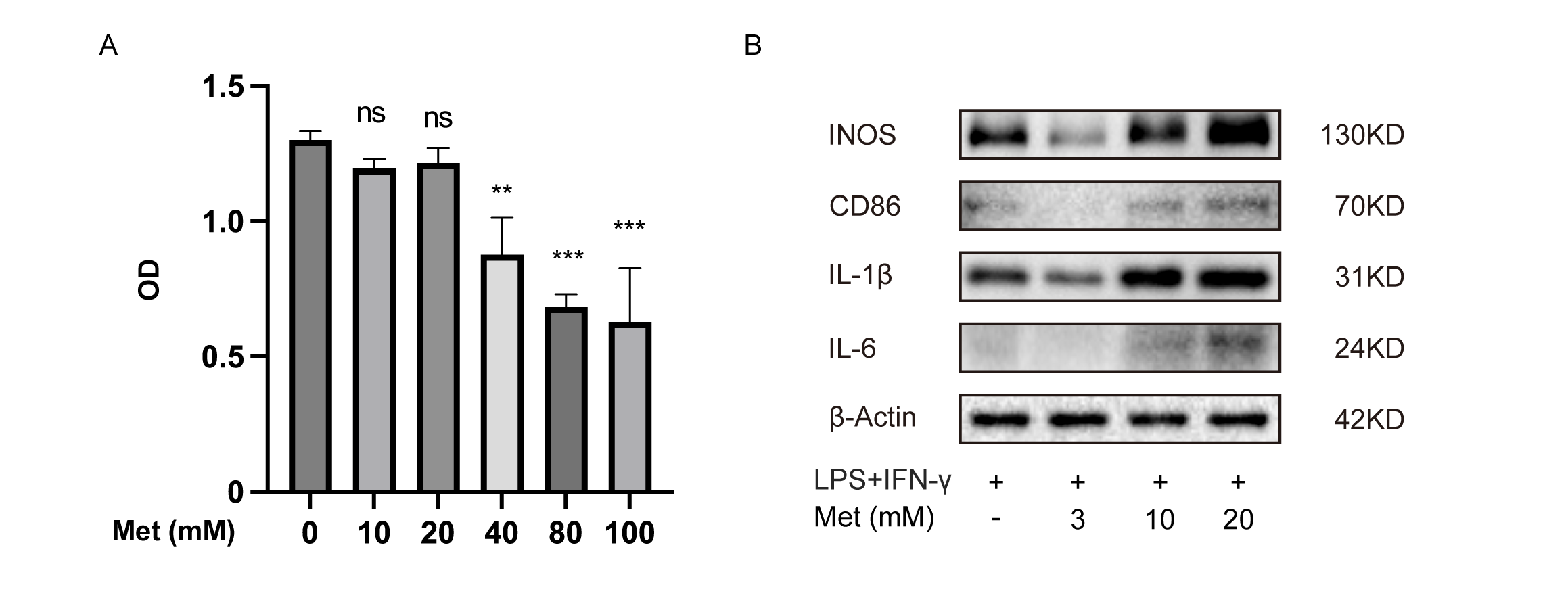

Supplement: Supplementary file 1 [file ijms-24-05355-s001.zip › ijms-2246825-supplementary.tif]
